# Systematic review of brain arteriovenous malformation grading systems evaluating microsurgical treatment recommendation

**DOI:** 10.1007/s10143-020-01464-3

**Published:** 2021-01-27

**Authors:** Basil E. Grüter, Wenhua Sun, Jorn Fierstra, Luca Regli, Menno R. Germans

**Affiliations:** 1 Department of Neurosurgery, University Hospital Zurich, University of Zurich, Frauenklinikstrasse, 10, 8091 Zurich, Switzerland; 2 Clinical Neuroscience Center, University Hospital Zurich, Zurich, Switzerland

**Keywords:** AVM, Grading, Microsurgical, Resection

## Abstract

When evaluating brain arteriovenous malformations (bAVMs) for microsurgical resection, the natural history of bAVM rupture must be balanced against the perioperative risks. It is therefore adamant to have a reliable surgical grading system, balancing these important factors. This study systematically reviews the literature in order to identify and assess the quality of grading systems with regard to microsurgical bAVM treatment. A systematic literature review was performed to provide an overview of all available bAVM grading systems relevant for microsurgical treatment evaluation and to assess the most comprehensive grading system specifically for each subgroup of bAVM (i.e., unruptured, ruptured, and posterior fossa). Screening of 865 papers revealed thirteen grading systems for bAVM microsurgical risk stratification. Among them, two systems were specifically developed for ruptured bAVM and one specifically for posterior fossa bAVM. With one system being fundamentally different for supratentorial bAVM, the remaining nine systems used the same parameters: “size,” “eloquence,” “venous drainage,” “arterial feeders,” “age,” “nidus compactness,” and “hemorrhagic presentation”. This study provides a comprehensive overview of all available bAVM grading systems relevant for surgical risk stratification. Furthermore, in the absence of a universal system appropriate to score all bAVMs, a workflow for selection of the best applicable scoring system in accordance with bAVM subgroups is presented.

## Introduction

Only few brain arteriovenous malformations (bAVMs) present with neurological symptoms; however, the annual risk of rupture is approximately 2.3%, [1] potentially leading to devastating intracranial hemorrhage with an estimated mortality of 10% and persisting morbidity of 30–50% [6, 9, 30]. Treatment modalities for bAVMs include endovascular, radiosurgical, and microsurgical procedures or a combination of them. The goal of treatment is complete bAVM eradication, which is the only way to prevent future hemorrhage. In many centers, treatment of bAVMs is discussed in interdisciplinary boards evaluating multimodal approaches. Nevertheless, microsurgical resection—often in combination with other treatments—is the modality of choice to achieve the highest rate of immediate and complete elimination of the bAVM nidus [8, 10, 40, 44]. The perioperative risks have to be taken into account for the decision-making process and treatment recommendation [5].

Since it is impossible to completely foresee the outcome of bAVM eradication, various grading systems for risk stratification in the different treatment modalities have been developed to predict the risk of complications and outcome. In recent years, several reviews of grading systems for endovascular and radiosurgical treatments have been published [15, 32, 33, 42]. However, no comprehensive review of microsurgical grading systems has been presented thus far.

The most often used grading systems for predicting the outcome after microsurgical resection are the Spetzler-Martin grade [38] and its refined version, the Spetzler-Ponce grade [39]—sometimes in combination with the supplementary grading scale [19]. Despite its widely accepted use, the Spetzler-Martin grade may not be the best predictor of outcome in certain subpopulations of bAVM patients, such as ruptured bAVM or bAVM of the posterior fossa. For these subgroups, other scoring systems have been designed that may better predict surgical risks or outcomes [2, 27, 29]. In order to create an overview of all existing grading systems with regard to microsurgical treatment of bAVM, we systematically reviewed the literature for all surgical bAVM grading systems and suggested a workflow for selecting the best applicable grading system for each bAVM subgroup.

## Materials and methods

### Search strategy

A literature search was performed on Pubmed/Medline, using the following search terms: (score OR scale OR grading) AND (arteriovenous malformation OR AVM OR arterio venous malformation) AND (surgery OR surgical). All studies published until July 19, 2019, were included. Duplicates were removed and two authors (BG and MG) independently screened titles, abstracts, and full texts for inclusion. Any discrepancy in study selection was discussed and decided upon consensus between the two authors. PRISMA guidelines (Preferred Reporting Items for Systematic Reviews and Meta-Analyses) [23] were strictly adhered to.

### Eligibility criteria

All studies in English with a primary description of a numeric score on surgical risk or clinical outcome in the context of microsurgical bAVM treatment were included. We excluded studies with a focus on endovascular, radiosurgical, or multimodal treatment scores. Surgical series identifying surgical risk factors or predictors of a good or poor outcome, respectively, without elaborating these factors into a numeric score were excluded too. Furthermore, we did not consider validation studies of pre-existing scores and non-original research papers such as comments, personal opinions, letters to the editor, or conference papers.

### Data extraction

The grading systems were analyzed for characteristics and details of the single factors that created the grading system and their relative weighting in the final system. Also size and characteristics of the study population were extracted, and type of outcome was assessed.

### Evaluation of the recommended system

Where applicable, studies were critically appraised according to the CHARMS checklist [24]. In order to find the best grading system for each subtype of bAVM, as a first step, the system with the highest prognostic value (as given in the original publication) was selected. The second step was confirmation in a validation study. Those systems with external validation were considered superior to those without. The last point taken into account was how complicated the presented system appeared to readers with regard to calculating and memorizing.

## Results

**Fig. 1:**
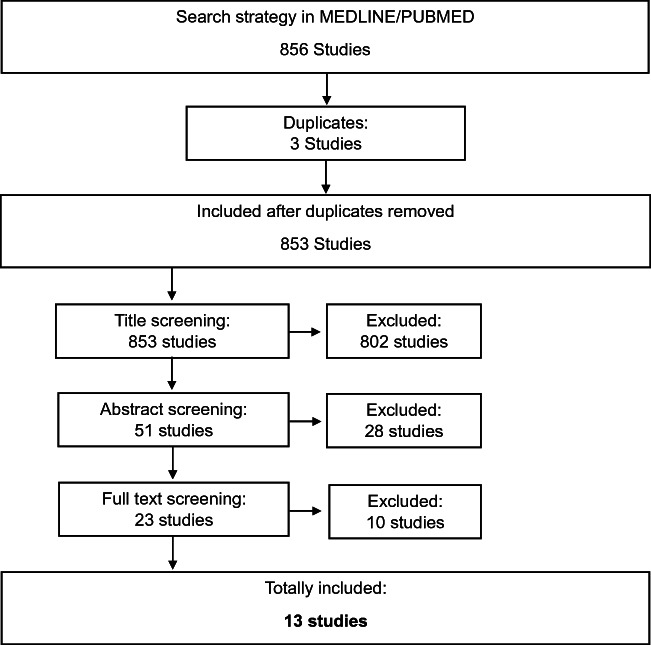
Flow chart of study selection. Of 856 studies identified by the search algorithm, thirteen studies eventually presented a score for risk prediction in bAVM surgery and were thus included for review in the present study

The search strategy revealed 865 studies of which thirteen studies met the inclusion criteria for further analysis (Fig. 1). Most of these studies (*n* = 10) were conducted in non-specified surgical populations, with two studies conducted in a population of exclusively ruptured bAVMs and one study on cerebellar bAVM only. Table 1 summarizes all thirteen studies included.

**Table 1:** Summary of grading systems predicting surgical risk or outcome in bAVM patients. Of the thirteen scores, two were developed specifically for ruptured bAVMs and one score for cerebellar bAVMs. Nine scores for unruptured supratentorial AVMs were essentially made from a pool of seven different factors. X in brackets (x) indicates that presence of the factor is mandatory for the score to be applied but the factor does not count in the score. AUC is given as indicated in the original publication. *AUC* area under curve, *GCS* Glasgow Coma Scale, *GOS* Glasgow outcome scale, *ICH* intracranial hemorrhage, *mRS* modified Rankin Scale, *n/a* not applicable

Author	Year	Score	Population	Detailed factors of grading system								Range	Type of outcome	AUC
				Size	Location/eloquence	Venous drainage	Arterial feeders	Age	Nidus compactness	Hemorrhagic presentation	Other specifics			n/a
Luessenhop [21]	1977		49 supratentorial bAVM				x					I-IV	Morbidity/mortality*	n/a
Shi [36]	1986		100 bAVM	x	x	x	x					1–4	Surgical result^†^	n/a
Spetzler [38]	1986	Spetzler-Martin	100 completely resected AVMs	x	x	x						I-V	Surgical result^‡^	n/a
Pertuiset [31]	1991	Operability Score	57 completely resected bAVM	x	x		x	x			• Straightening of feeding artery • Sectorization • Vascular autoregulation • Circulatory velocity • Brain tissue cellular steal • Previous rupture • Malformations of vital organs • Associated disease	3–69	Surgical result^§^	n/a
Tamaki [41]	1991	Angiographic grading system	151 bAVM	x	x		x					0–4	Clinical grading^||^ and post-op Karnofsky	n/a
Höllerhage [13]	1992		93 bAVM				x				• Clinical presentation upon admission	1–7	Outcome scale^#^	n/a
Spears [37]	2006	Toronto model	233 bAVM		x	x			x			0–9	mRS, GOS	0.79
Lawton [19]	2010	Supplementary grading scale	300 bAVM					x	x	x		1–5	mRS	0.78
Spetzler [39]	2011	Spetzler-Ponce	1476 patients from 7 surgical series	x	x	x						A-C	Various**	0.71
Appelboom [2]	2011	ICH score	84 patients with ruptured bAVM					x		(x)	• GCS • ICH volume • Intraventricular blood • Infratentorial origin of ICH	0–6	mRS	0.89
Neidert [27]	2016	AVICH score	67 patients with ruptured bAVM	x	x	x		x	x	(x)	• GCS • ICH volume • Intraventricular blood	2–13	mRS	0.84
Jiao [14]	2018	HDVL grading system	201 surgically treated bAVMs			x			x	x	• Lesion to eloquence distance	1–6	mRS	0.82
Nisson [29]	2019		120 cerebellar bAVM			x		x			• Neurological status prior to surgery • Emergency surgery	1–3	mRS	0.74

*Luessenhop refers to morbidity/mortality in general, without further specification or application of an outcome score

^†^Shi subdivides operative results in “excellent” (normal neurological function), “good” (maintenance of preoperative neurological function), and “poor (new neurological deficits or increase or preoperative deficits)

^‡^In the Spetzler-Martin paper, bAVM grades were correlated with deficits (no/minor/major) and death in the corresponding group. As major deficits, they listed hemiparesis, increase in aphasia, homonymous hemianopsia, and severe neurological deficits.

^§^Pertuiset specified results from surgery into “no deficit,” “minor deficit,” “permanent deficit,” and “death”

^||^Clinical grading by Tamaki was defined as “excellent” (no deficits), “good” (mild deficits), “fair” (moderate deficits), “poor” (severe deficits) and death

^#^The outcome scale of Höllerhage differentiates grade I (no deficit, no seizure), grade II (discrete deficits such as slight sensory deficits or discrete palsies without seizures), grade III (distinct palsies or seizures or psychic deficits but still independent life), grade IV (severe deficits, dependent on care), grade V (death)

**The seven surgical series analyzed by Spetzler and Ponce used different scales for measurements of outcomes, among them: “minor deficit – major deficit – death”; “excellent – good – fair – poor”; “unchanged or improved vs permanent neurological deficits”; “any worsening of patients” and modified Rankin Scale

All scoring systems predominantly contained seven factors: bAVM size, nidus location (or so-called eloquence), venous drainage, arterial feeders, nidus compactness, hemorrhagic presentation, and patient age. The factors which were most often included as a contributing factor to risk assessment were location/eloquence of the AVM [27, 31, 36–39, 41] and venous drainage [14, 27, 29, 36–39] (7/13 scores, 54%), followed by nidus size in 6/13 scores (46%) [27, 31, 36, 38, 39, 41]. Consideration of arterial feeders was popular in historical studies [13, 21, 31, 36, 41] but is not a contributing factor in any scores presented after 1992. On the other hand, observations of nidus compactness and hemorrhagic presentation were only taken into consideration in scores developed from 2006 onwards [2, 14, 19, 27, 37]. The two scores of Höllerhage [13] and Nisson [29] put an additional emphasis on clinical presentation before surgery. The two scores for ruptured bAVM only [2, 27] include Glasgow Coma Scale (GCS) score on admission, intraventricular blood, hemorrhage volume, and age as relevant factors in predicting outcome.

The scores included 3tier [29, 39], 4tier [21, 36, 41], 5tier [19, 38], 6tier [14], 7tier [2, 13], 10tier [37], and 12tier [27] system, and the most elaborate 66-point system by Pertuiset [31]. In the latter, the score is subsequently subdivided into five categories. The scores of Luessenhop [20], Shi [36], and Pertuiset [31] equally weighed each contributing factor. All other scoring systems weighed certain factors stronger than others, such as nidus size [27, 38, 39], age [19, 27], or eloquence [14, 37].

All scoring systems presented after 1992 measured outcome with the modified Rankin scale (mRS), except for the Spetzler-Ponce grading system [39]. This system was verified in various cohorts to which different systems for outcome measurements were applied. All papers using the mRS-score set a cut-off for good outcome at a mRS of ≤ 2. Historical series often used self-defined scales for outcome measurements (see Table 1 for a detailed description of outcome measurements).

## Discussion

Our systematic literature review identified thirteen scoring systems for surgical risk stratification in bAVM surgery. Of these thirteen systems, six should be regarded in a historical context, four are up-to-date to grade supratentorial bAVMs, two are specifically developed for ruptured bAVMs, and one score is presented exclusively for infratentorial bAVMs. All of them but one consist of a selection from the same seven factors (size, location/eloquence, venous drainage, arterial feeders, age, nidus compactness, and hemorrhagic presentation), which are combined and weighed differently for each grading system and sometimes refined with more specific extra variables.

### Historical grading systems

One of the problems of comparing older grading systems to more modern ones is that older systems did not use an objective measurement for surgical outcome. Despite the first introduction of the ranking scale being in 1957 [34], and its modification for better interobserver reliability in 1988 [45], Spears was the first author to use mRS for outcome assessment in his grading system in 2006. Earlier studies used self-defined methods for measurement of outcome (see Table 1 for detailed description). Furthermore, study populations were highly selective and often the own series of the presenting surgeon: the system from Lussenhop et al. [21] was developed after studying 300 angiograms of bAVM patients, but the authors demonstrated the clinical usefulness of their system based on the postoperative results of a series of 49 patients only. Shi [36] applied his system retrospectively to 100 patients whose bAVM had been completely excised in order to correlate his operative results with the grades assigned. Likewise, in the original Spetzler-Martin publication, Spetzler applied his presented system retrospectively to his own series of a hundred completely resected AVMs [38]. The operability score, introduced by Pertuiset [31], was applied to 57 cases out of 295 supratentorial bAVMs treated by one author. This system scores multiple anatomical, hemodynamic, and clinical factors and allocates a value of 3–69 points to each bAVM. As accurate as it may be in risk prediction, this grading system lacks one of the fundamental properties of a successful score: easy to memorize and therefore suitable for routine clinical application. By contrast, the study of Tamaki is based on “long-term” follow-up of 151 patients [41]. Long-term is defined as time from treatment to the last follow-up. The study lacks an exact statement of duration but was published in 1991 and indicates recruitment between 1970 and 1990; thus, long-term may theoretically be anywhere between 1 and 21 years. The study population consisted of five treatment categories: complete resection, subtotal resection, partial resection, other surgical interventions other than bAVM resection (such as hematoma removal, ventricular shunt or feeder clipping before radiation) and conservative treatment. Lastly, Höllerhage et al. developed his grading system with 93 patients who had an angiogram and underwent surgery for bAVM. This meant explicitly that patients whose angiogram was performed in a different center were excluded and were not available to the study team. The authors performed a stepwise multiple regression of all the factors influencing outcome.

With the exception of the study from Spetzler, considerations on arterial feeders have been the backbone of the older grading systems in the era of early angiography. While this feature is still the most relevant in endovascular grading systems [33], none of the grading systems presented after 2006 takes arterial feeders into account as a factor. Nevertheless, recent surgical papers also report on worse outcome associated with deep perforating artery supply [7] or, more specific, increased risks related to lenticulostriate arterial supply [25]. The main challenge of these deep arteries is their location on the deep side of the bAVM, behind the nidus. As such, they are not visible to the neurosurgeon. Manipulation of the bAVM also harbors a risk of rupture and bleeding in the depth, which may be challenging to control. Additionally, these deep arteries are difficult to obliterate because they are not easily coagulated and retract, which proposes the risk of digging into the deep white matter.

### Modern supratentorial grading systems

The original Spetzler-Martin grading system [38] was revised into the Spetzler-Ponce [39] system in 2011. This revision not only included a summary of the former five (I-V) into three (A, B, C) risk groups, but the grading system was also verified in 1476 patients from seven independent surgical series. Later, this grading system was verified in numerous cohorts and was successfully applied to many surgical series [4, 18, 26, 35]. The Spetzler-Ponce system is based on the same three determinants “size,” “eloquence,” and “venous drainage” as the original Spetzler-Martin system. While “size” and “deep venous drainage” (defined as any or all of the drainage through deep veins such as the internal cerebral veins, the basal veins, or precentral cerebellar vein) [38] are well accepted as factors to complicate surgery, “eloquence” may be a more disputed one. Eloquence is defined as the sensorimotor, language and visual cortex, and a list of deeper structures, namely the thalamus and hypothalamus, the internal capsule, the brain stem, the cerebellar peduncles, and the deep cerebellar nuclei [38]. Notably, not all studies completely applied this definition; for example Mascitelli et al. found sensorimotor and language, but not visual eloquence, to be associated with worse clinical outcome [22].

The Toronto model [37] was developed in 233 consecutive patients who underwent bAVM surgery (out of 1058 bAVM patients treated during the study period) at the same institution. However, 38.6% underwent at least one embolization as part of their treatment, 5.1% underwent radiation, and 4.3% had both embolization and radiation prior to surgery. Where the first 175 patients were used to derive the grading system, the last 58 were used to validate it. With regard to content, the grading system is similar to the Spetzler-Ponce system. However, the authors replaced “size” with “nidus compactness” and weighed the three factors with odds ratios (eloquence 4, diffuseness 3, deep venous drainage 2). Diffuse nidus is a condition that makes surgical resection considerably more challenging and is associated with worse outcomes after microsurgical resection [7]. The rounded odds ratios form a weighed 9-point prediction model specifically designed for early and permanent neurological deficits. Its relatively cumbersome calculation, the lack of external validity, and its relative similarity to the much better known Spetzler-Ponce system may be reasons why the Toronto grading system never became popular for clinical or scientific work.

The parameter “nidus configuration” was also incorporated in the supplementary grading scale [19] of Lawton et al. together with “age” and “hemorrhagic presentation.” This grading system was designed to increase accuracy in predicting neurological outcome after bAVM surgery and to refine patient selection. The aim was to supplement, rather than replace, the pre-existing Spetzler-Martin grading system. Outcome measurements were performed by a nurse clinician, under the supervision of an independent neurologist. Lawton tested the model in his own series of 300 microsurgically treated patients. However, a 10-fold cross validation of the system confirmed similar results. Later on, an international validation study of 1009 surgical patients (including the 300 original patients +117 patients treated in that same institution afterwards and an additional 592 patients from three other countries) demonstrated that the supplemented Spetzler-Martin score indeed performed better than the original Spetzler-Martin score for both medium- and long-term follow-up [17].

Likewise, Jiao et al. presented a grading system that was initially designed to supplement the established Spetzler-Martin system [14]. The study was conducted retrospectively on 201 consecutive patients who underwent microsurgical resection of their bAVM in a single institution. Two experienced neurosurgeons collected the clinical information from the prospectively collected database and medical records. All patients had to undergo functional MRI (fMRI) and diffusion tensor imaging (DTI). The system scores “hemorrhagic presentation”, “diffuseness of the nidus,” “deep venous drainage” with 1 point each, and “lesion-to eloquence distance (LED)” with 1–3 points. Interestingly, the authors found a higher predictive value of their system when applied on its own, without inclusion of “size” and “eloquence,” as their supplemented full grading system suggested. Furthermore, they found the LED, determined by fMRI and DTI, to be a single significant predictor of operative risk. Not only did this study report a benefit of modern imaging techniques in bAVM surgery, also DTI of the long association bundles, [3] high-resolution three-dimensional multifusion imaging (CT-MRI fusion with combined surface and volume rendering) [46], and MRI fusion with time-resolved (so called 4D) DSA [43] have been reported to be helpful for preoperative planning and intraoperative navigation and eventually to contribute to better surgical outcome.

### Systems for grading of ruptured AVMs

When it comes to scoring of ruptured bAVMs, two systems have been suggested: the original ICH grading system [2] and the AVICH grading system [27]. The ICH grading system was developed for risk stratification of non-traumatic intracranial hemorrhage (ICH) [11]. Therefore, the authors tested if it also predicts outcome of hemorrhage related to ruptured bAVM. In this study, 84 patients were included (out of 91 consecutive patients diagnosed with a bAVM, 7 excluded due to missing data). From the study population, only 31% received direct surgical treatment, whereas 45% received a combination of embolization and resection, 12% underwent radiosurgical procedures, 2% combined embolization and Gamma Knife, and 9.5% was managed conservatively. Given that the etiology of non-traumatic ICH is predominantly hypertensive, followed by drug-related [16], Neidert et al. observed that clinical outcome of patients with spontaneous ICH was worse than in bAVM-related ICH. The authors hypothesized that the ICH score was insufficient to reflect the different pathophysiology of bAVM-related ICH [27]. In order to overcome this problem, they merged the existing ICH score with the supplemented Spetzler-Martin grading system and thereby created the AVICH grading system. This system was primarily established in a single center cohort, consisting of 67 consecutive patients either pretreated before presentation for their bAVM or newly diagnosed with a bAVM and an associated ICH. As a next step, external validation in an international multicenter study cohort revealed superiority of the AVICH score over the ICH score [28]. In this validation study, eleven centers provided data on a total of 325 patients. The study calculated the Spetzler-Martin grade, the supplemented grading score, the ICH score, and the AVICH score for the entire study population. Despite the fact that the ICH score showed a higher AUC (0.891) in the original publication than the AVICH score (0.842), the external validation revealed superiority of the AVICH score over the ICH score [28]. Whereas the AVICH grading system may be more precise in outcome prediction than the original ICH score, it is rather complex with eight factors to score (among them an emphasized weighing of size and age) and a range of eleven.

### Grading for AVMs of the posterior fossa

There is a growing body of evidence that shows that infratentorial bAVMs behave more aggressively than their supratentorial counterparts, with an estimated 5-year rupture rate as high as 12% [12]. In consequence, posterior fossa bAVMs are more likely to rupture and patients with these lesions are more likely to present with symptomatic hemorrhage. The grading system by Nisson et al. deals exclusively with bAVM of the posterior fossa [29]. The study originated in the USA and was co-authored by Spetzler and Lawton. Out of 125 patients with cerebellar bAVMs, 120 were treated microsurgically (in two tertiary medical centers) and included in the study. Data were collected retrospectively by independent research faculty members. The authors did not elaborate on pre-treatment. The cohort consisted for 13% of children and 71% of patients presented with a hemorrhage. Furthermore, the authors found deep venous drainage, older age, poorer neurological status on admission, and need for emergency surgery to be the relevant risk factors for poor outcome. Interestingly, cerebellar bAVM size did not correlate with outcome. The authors found a strong association of emergency surgery with poor outcome and concluded that this is a result of a hematoma with mass effect in close proximity to vital structures. They handled this increased risk by weighing “emergency surgery” double in their scoring system. The grading system continues to lack external validation, but it is the only system specifically addressing risk stratification for surgical treatment of infratentorial AVMs.

### What makes a grading system a good one?

The requirements for a good grading system are manifold. First, it must have a high accuracy in predicting surgical risk. Predictive accuracy describes a combined function of sensitivity and specificity. It can be calculated with receiver operating characteristic (ROC) analyses. In this statistical testing, the area under the curve (AUC) indicates the probability that a patient with a poor outcome after surgery would have a higher value on the score than a patient with a good outcome. Thereby, an AUC of 1 reflects perfect discrimination whereas an AUC of 0.5 reflects that results are based purely on coincidence. In a clinical context, discrimination levels > 0.7 are considered acceptable. The available AUC values for grading systems in our review are provided in Table 1. Secondly, a grading system has to confirm its prognostic value in a different, independent cohort (so-called external validation). From the described grading systems, the Spetzler-Ponce score, the supplementary grading scale, and the AVICH score were successfully validated in multicenter cohorts [17, 28, 39]. Finally, in order to establish routine clinical application of a grading system, it must be easy to memorize and quick to calculate. A high number of parameters, elaborative assessment of the parameter (i.e., post-processed images such as fMRI or DTI), or complex mathematical weighing of the individual parameters may all lead to greater accuracy of a grading system. However, these issues create a system which is too complicated to use in clinical routine. The result of an ideal grading system should essentially dichotomize between surgical candidates and non-surgical candidates. Therefore, a “1 or 0” principle or an “A, B, C” categorization may even be more helpful than assignment to a broad range of different risk categories. This is one of the strengths of the Spetzler-Ponce score and may be a reason why the Toronto model never established itself as a clinical standard.

### Summary of most important grading systems

**Fig. 2:**
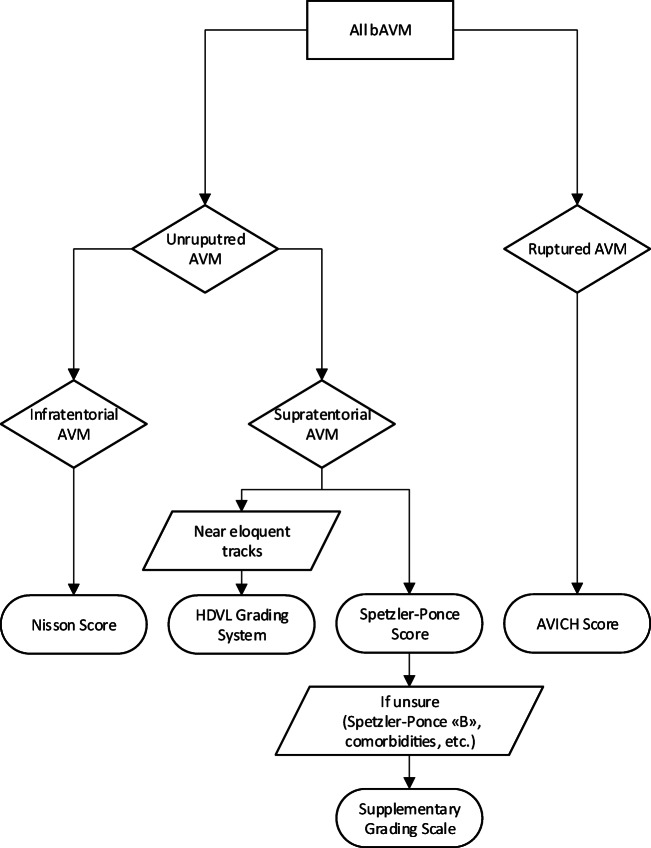
Flow chart giving an overview of possible grading system in accordance with type of bAVM. Spetzler-Ponce and supplemented Spetzler-Ponce scores have a high predictive value and were approved in external validation. Likewise, the AVICH score proved to be a reliable predictor of surgical risk in an international multicenter validation study. The HDVL grading system still lacks external validation, but the approach to include fiber tracking may be trendsetting for risk stratification in certain bAVMs near eloquent areas. Lastly, the Nisson score was developed specifically for AVMs of the posterior fossa

**Fig. 3:**
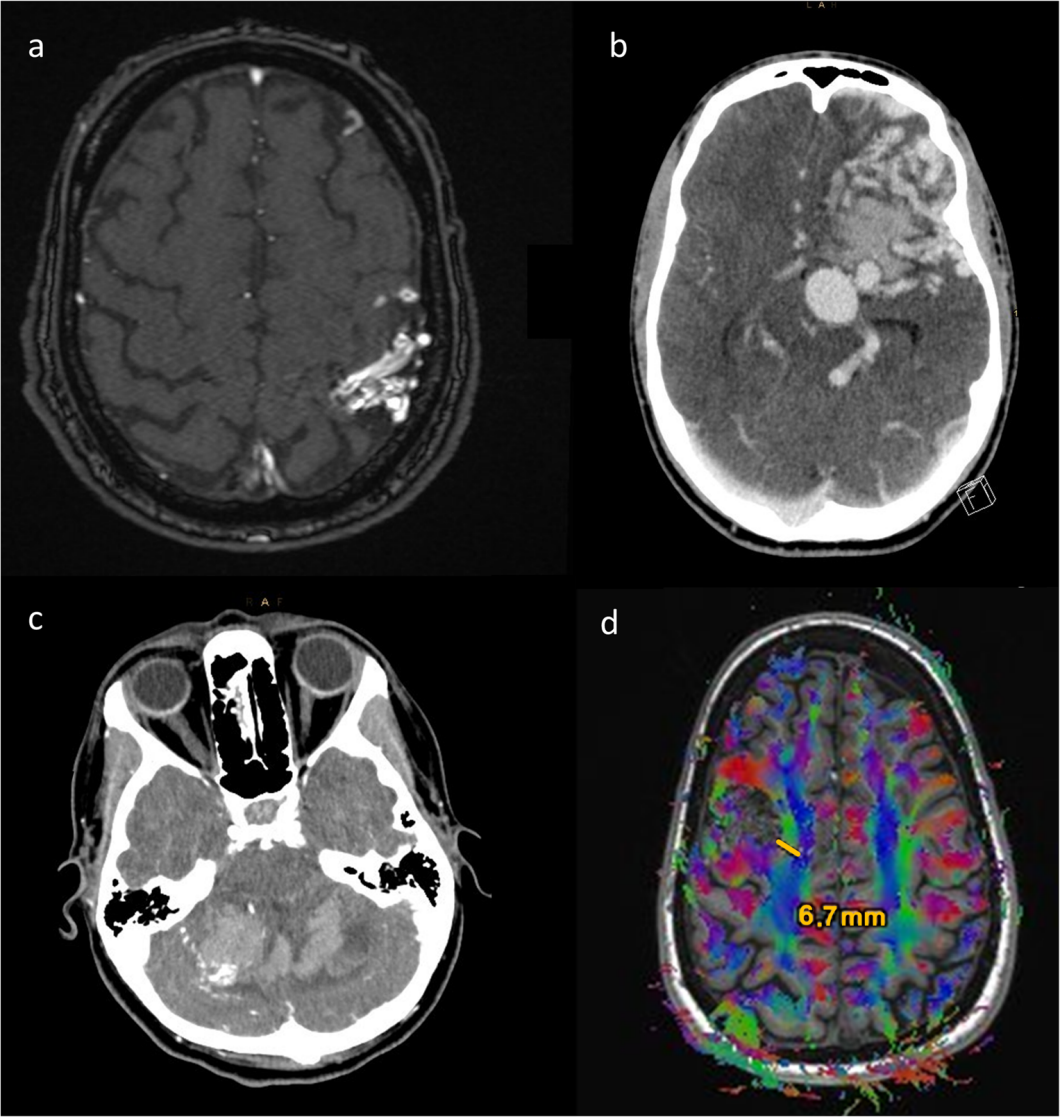
Illustrative cases of different types of AVMs and corresponding grading system. **a** TOF-angiography of a 60-year-old patient showing a non-ruptured central AVM, measuring 21 mm, with superficial venous drainage and compact nidus. The Spetzler-Ponce system scores this lesion as A (1 point of for size and 1 for eloquence), the supplementary grading scale adds 3 points (for age > 40). **b** Ruptured AVM in the left frontal lobe of a 24-year-old patient with GCS 7 at presentation. This lesion is scored 10 according to the AVICH score (1 point each for deep drainage, eloquence, GCS, intracerebral hemorrhage volume (44 cc), and presence of intraventricular hemorrhage, 2 points were given for age of the patient, and 3 points for size (62 mm) of the AVM. The nidus appeared compact). **c** Ruptured cerebellar AVM in a 23-year-old patient with GCS 13 on admission who was neurologically intact upon emergency presentation. She underwent emergency surgery (decompression within 24 h), followed by AVM resection 3 weeks later. Nisson score reveals a grade I lesion in this patient, with only 1 point for emergency surgery. **d** MR tractography of a 49-year-old patient with an unruptured AVM in the right frontal lobe, adjacent to the cortico-spinal tract. His HDVL grade scores 3 (no preoperative hemorrhage and LED 6.7 mm)

Because this review showed that none of the existing grading systems is universal in their application, we created a flowchart that suggests an adequate grading system in accordance with the subtype of bAVM. This may help to provide support in deciding which grading system to use for an individual patient. Still, treatment will be based on an individualized risk assessment for every patient and it is left at the discretion of the treating surgeon to decide which tools to use for risk stratification. Grading systems with external validation were considered superior to those without. Based on the current literature, we suggest the application of the (supplemented) Spetzler-Ponce score for unruptured supratentorial bAVM. The HDVL score [14] still lacks external validation but it may be helpful for lesions near eloquent fiber tracks. The AVICH score seems to be the most precise grading system for surgical risk stratification in ruptured bAVM. Finally, the Nisson score is the only system developed specifically for infratentorial bAVM. The flow chart in Fig. 2 gives an overview of possible grading system in accordance with type of bAVM, and Fig. 3 shows exemplary cases graded according to the most adequate system.

### Limitations

It must be emphasized that heterogeneity of data between different studies, for instance outcome measurements, makes it difficult to compare these studies directly. However, nearly all of the studies published in the last decade used the mRS for outcome measurement, except the Speztler-Ponce publication. In this study, several outcome measurements were applied, including the mRS. Furthermore, the cohorts in which the grading systems were developed were heterogenous for various patient characteristics, for instance pre-treatment (embolization, radiosurgery) and hemorrhagic presentation. However, this probably reflects the reality of clinical routine. As bAVMs are a relatively rare condition, a grading system should aim to be as universal as possible. Finally, none of the presented grading systems is perfect and clinical judgment still remains an important factor for the decision to resect the bAVM.

## Conclusions

This study provides a comprehensive overview of all available bAVM grading systems relevant for surgical risk stratification. In the absence of a universal system appropriate to score all bAVMs, we suggest the application of the (supplemented) Spetzler-Ponce score for supratentorial bAVMs, the HDVL-grading score for bAVMs near eloquent tracks, the AVICH score for ruptured bAVMs, and the Nisson score for infratentorial bAVMs.

## Data Availability

The authors confirm that the data supporting the findings of this study are available within the article.
